# Mitochondrial dysregulation occurs early in ALS motor cortex with TDP-43 pathology and suggests maintaining NAD^+^ balance as a therapeutic strategy

**DOI:** 10.1038/s41598-022-08068-5

**Published:** 2022-03-11

**Authors:** Mukesh Gautam, Aksu Gunay, Navdeep S. Chandel, P. Hande Ozdinler

**Affiliations:** 1 Department of Neurology, Feinberg School of Medicine, Northwestern University, 303 E. Chicago Ave, Chicago, IL 60611 USA; 2 Department of Medicine, Biochemistry and Molecular Genetics, Northwestern University Feinberg School of Medicine, Chicago, IL 60611 USA; 3 Center for Molecular Innovation and Drug Discovery, Center for Developmental Therapeutics, Chemistry of Life Processes Institute, Northwestern University, Evanston, IL 60611 USA; 4 Mesulam Center for Cognitive Neurology and Alzheimer’s Disease, Feinberg School of Medicine, Northwestern University, Chicago, IL 60611 USA; 5 Feinberg School of Medicine, Les Turner ALS Center at Northwestern University, Chicago, IL 60611 USA

**Keywords:** Cellular neuroscience, Amyotrophic lateral sclerosis

## Abstract

Mitochondrial defects result in dysregulation of metabolomics and energy homeostasis that are detected in upper motor neurons (UMNs) with TDP-43 pathology, a pathology that is predominantly present in both familial and sporadic cases of amyotrophic lateral sclerosis (ALS). While same mitochondrial problems are present in the UMNs of ALS patients with TDP-43 pathology and UMNs of TDP-43 mouse models, and since pathologies are shared at a cellular level, regardless of species, we first analyzed the metabolite profile of both healthy and diseased motor cortex to investigate whether metabolomic changes occur with respect to TDP-43 pathology. High-performance liquid chromatography, high-resolution mass spectrometry and tandem mass spectrometry (HPLC–MS/MS) for metabolite profiling began to suggest that reduced levels of NAD+ is one of the underlying causes of metabolomic problems. Since nicotinamide mononucleotide (NMN) was reported to restore NAD^+^ levels, we next investigated whether NMN treatment would improve the health of diseased corticospinal motor neurons (CSMN, a.k.a. UMN in mice). prpTDP-43^A315T^-UeGFP mice, the CSMN reporter line with TDP-43 pathology, allowed cell-type specific responses of CSMN to NMN treatment to be assessed in vitro. Our results show that metabolomic defects occur early in ALS motor cortex and establishing NAD^+^ balance could offer therapeutic benefit to UMNs with TDP-43 pathology.

## Introduction

Mitochondria are best studied for their ability to generate energy, but they are also essential for lipid homeostasis, ensuring proper Ca^+2^ storage inside the cells, and maintaining metabolomic balance^1–5^. With their multiple important functions, they serve as one of the key organelles for cellular health. Therefore, it is no surprise that mitochondrial dysfunction emerges as one of the common causes of neurodegeneration in many different diseases, and ALS is no exception.

TDP-43 pathology is one of the common proteinopathies observed in a large spectrum of ALS patients, including familial, sporadic and ALS with frontotemporal lobar dementia (ALS/FTLD)^6–9^. Even though some patients have mutations in *TARDBP*, the gene that codes for TDP-43 protein, not all TDP-43 pathologies are associated with the mutation^10^, and not all protein accumulations occur in the cytoplasm, some are detected in the nucleus—^11,^^12^. In fact, most of the patients with TDP-43 pathology do not have mutations in their *TARDP* gene, and yet have protein accumulations containing phosphorylated TDP-43^13^, and mostly ubiquitinated protein aggregates^10,14,15^. One of the interesting findings related to the neurons with TDP-43 pathology is the mitochondrial defects observed at a cellular level^16–18^. Such mitochondrial defects are also present in the upper motor neurons (UMNs) that are diseased in ALS. Regardless of species, the UMNs of a broad spectrum of ALS patients and corticospinal motor neurons (CSMN; a.k.a UMN in mice) of well characterized mouse models of TDP-43 pathology, both display prominent mitochondrial defects in a cell-type specific manner^16^.

There is an immense effort to reveal the underlying causes of TDP-43 pathology and the downstream cellular events that are perturbed in diseased neurons. Since mitochondrial defects have been broadly observed in many different diseases in which TDP-43 pathology is commonly detected, and because mitochondria are crucially important for maintaining metabolomic homeostasis, one of the key questions that remains unanswered is: “What are the metabolomic changes that occur with respect to TDP-43 pathology?” We investigated the metabolomic perturbations that occur in the motor cortex of prp-TDP-43^A315T^ mice, a mouse model which recapitulates many aspects of TDP-43 pathology observed in ALS and ALS/FTLD patients^19^. An early symptomatic stage of the disease was selected to investigate whether metabolomic perturbations begin to occur early in the motor cortex with TDP-43 pathology.

Our findings began to reveal reduced ATP production as well as perturbations in the balance of key metabolites, such as NAD^+^, GSH (glutathione), and PEP (phosphoenol pyruvate). We thus tested the hypothesis that increasing NAD^+^ levels would help restore balance and improve the health of UMNs diseased due to TDP-43 pathology. NAD^+^ plays a crucial role in several physiological conditions such as neurological disorders, diabetes, aging, oxidative stress and neurodegenerative diseases^20–26^. Since NMN (nicotinamide mononucleotide) was previously reported to increase the levels of NAD^+^ by acting as the precursor for NAD^+^^27^, we investigated whether NMN treatment would have therapeutic impact on diseased UMNs.

In an effort to bring cellular clarity to our analyses, and to investigate UMNs direct response to treatment, we took advantage of the prpTDP-43^A315T^-UeGFP mice, a CSMN reporter line in which diseased UMNs in mice that have TDP-43 pathology retain their fluorescence both in vivo and in vitro. Our results not only reveal early metabolomic problems in the motor cortex, but also suggest that maintaining NAD^+^ balance could offer a therapeutic treatment strategy for diseased UMNs with TDP-43 pathology.

## Results

### Metabolite profiling in the motor cortex with TDP-43 pathology reveals energy deficiency and oxidative stress

**Figure 1:**
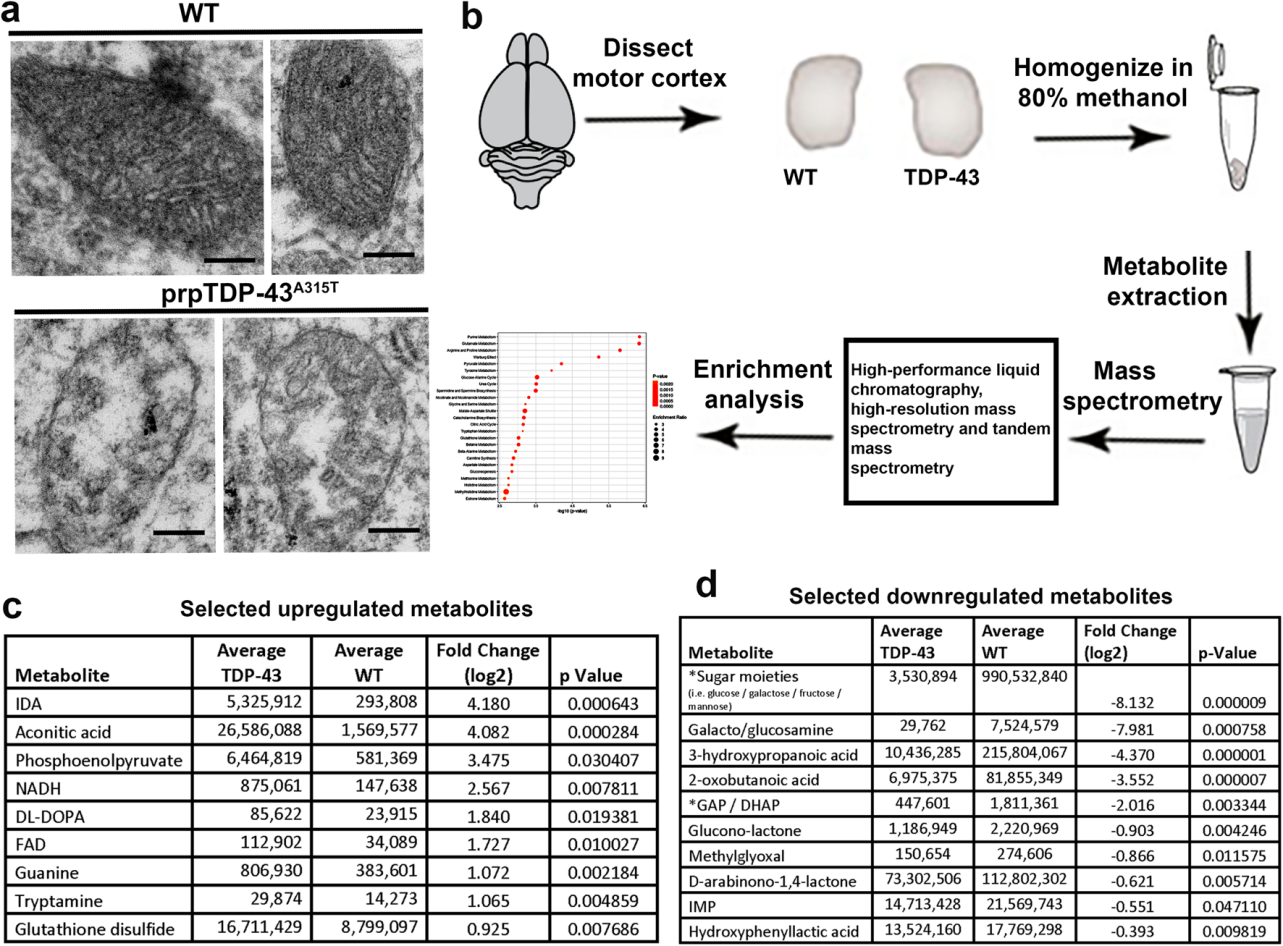
Metabolite profiling of motor cortex revealed dysregulated metabolite due to TDP-43 pathology. (**a**) representative electron microscopy images of mitochondria in WT healthy CSMN and in CSMN that are diseased due to TDP-43 pathology (**b**) schematic representation of workflow of study design. (**c**) list of selected upregulated metabolites. (**d**) List of selected downregulated metabolites. Scale bar = 200 nm.

We previously identified major mitochondrial problems, especially within the corticospinal motor neurons (CSMN) of prpTDP-43^A315T^ mice^16^ (Fig. 1a). Mitochondrial problems were present as early as P15 (postnatal day 15)^17^, and they were also observed in the upper motor neurons (UMNs) of a broad spectrum of ALS patients with TDP-43 pathology^16^. Since mitochondrial problems begin to occur early and because they are shared among species, we first investigated potential changes in metabolite dynamics, especially in the motor cortex of prpTDP-43^A315T^ mice, which closely recapitulates ALS pathology in patients^16,17,19^. Primary motor cortices of 90 days old (P90) prpTDP-43^A315T^ and healthy wildtype (WT) control mice were dissected out and metabolites were isolated by methanol extraction method followed by high performance mass spectrometry profiling of metabolites (Fig. 1b). We chose P90 as the time of investigation, because this is a symptomatic stage with profound CSMN loss, and it coincides to the disease stage, when patients show symptoms and TDP-43 pathology begins to occur. Analyses of metabolites resulted in the identification of distinct set of metabolites (Supplementary Table 1), some of which are upregulated (Fig. 1c), and some downregulated (Fig. 1d) with respect to healthy WT control motor cortex.

**Figure 2:**
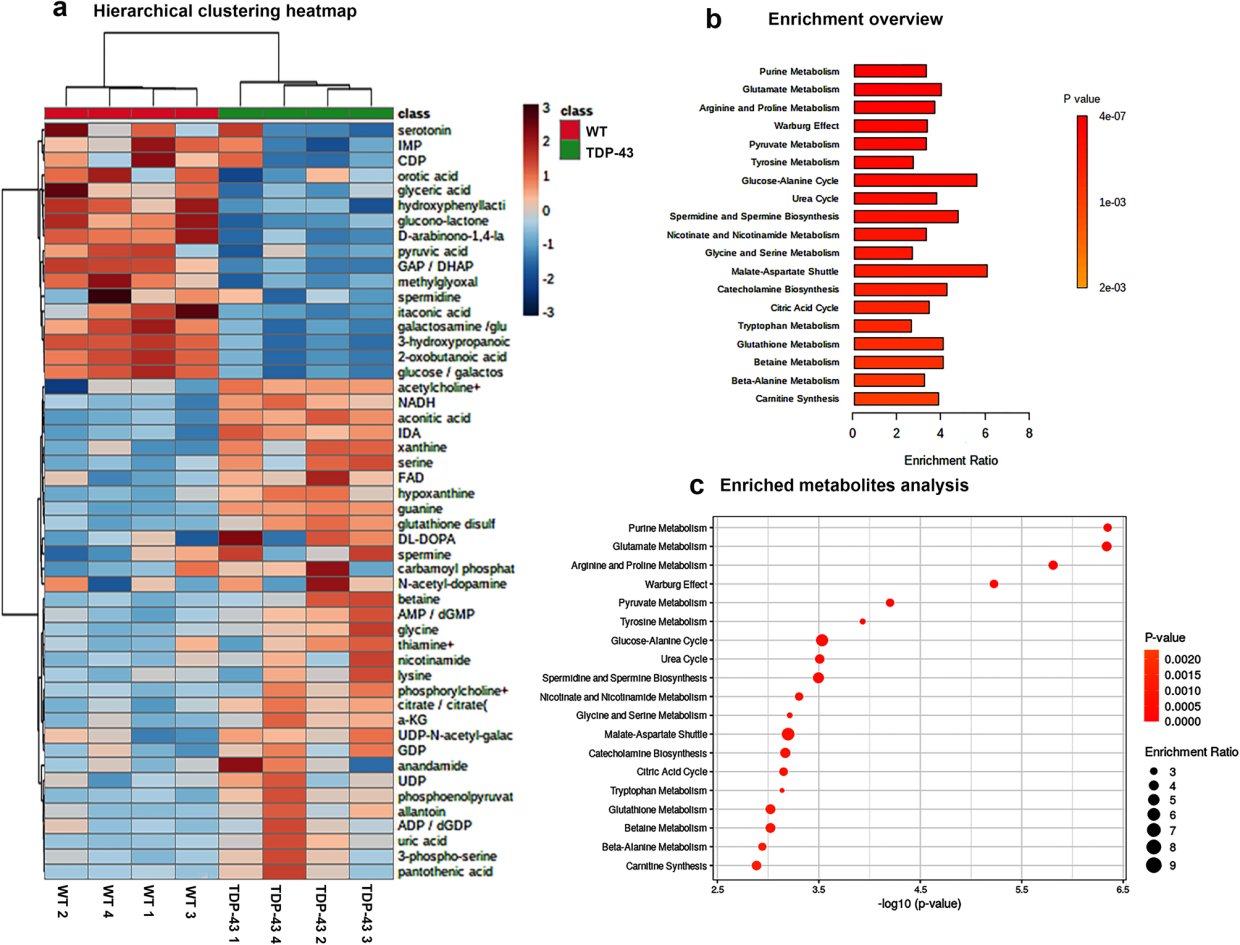
Enrichment analysis of metabolites. (**a**) Hierarchical clustering heat map of top 50 differentially present metabolites, (**b**) overview of enriched metabolites, and (**c**) analysis of enriched metabolites.

Enrichment analysis was performed using Metaboanalysis software^28^. Heatmap of hierarchical clustering analysis of top 50 highly enriched metabolites revealed differential presence of key metabolites (Fig. 2a). Pyruvate metabolism, tyrosine and nicotinamide metabolism, glycine and serine metabolism, citric acid cycle, glutathione metabolism, and carnitine synthesis were among important metabolite pathways that were different between healthy and diseased motor cortex (Fig. 2b,c). Glutamate is precursor for glutathione biosynthesis. Purine and glutamate metabolism were the top hits, suggesting perhaps ATP and glutathione biology are impacted. (Fig. 2a–c).

**Figure 3:**
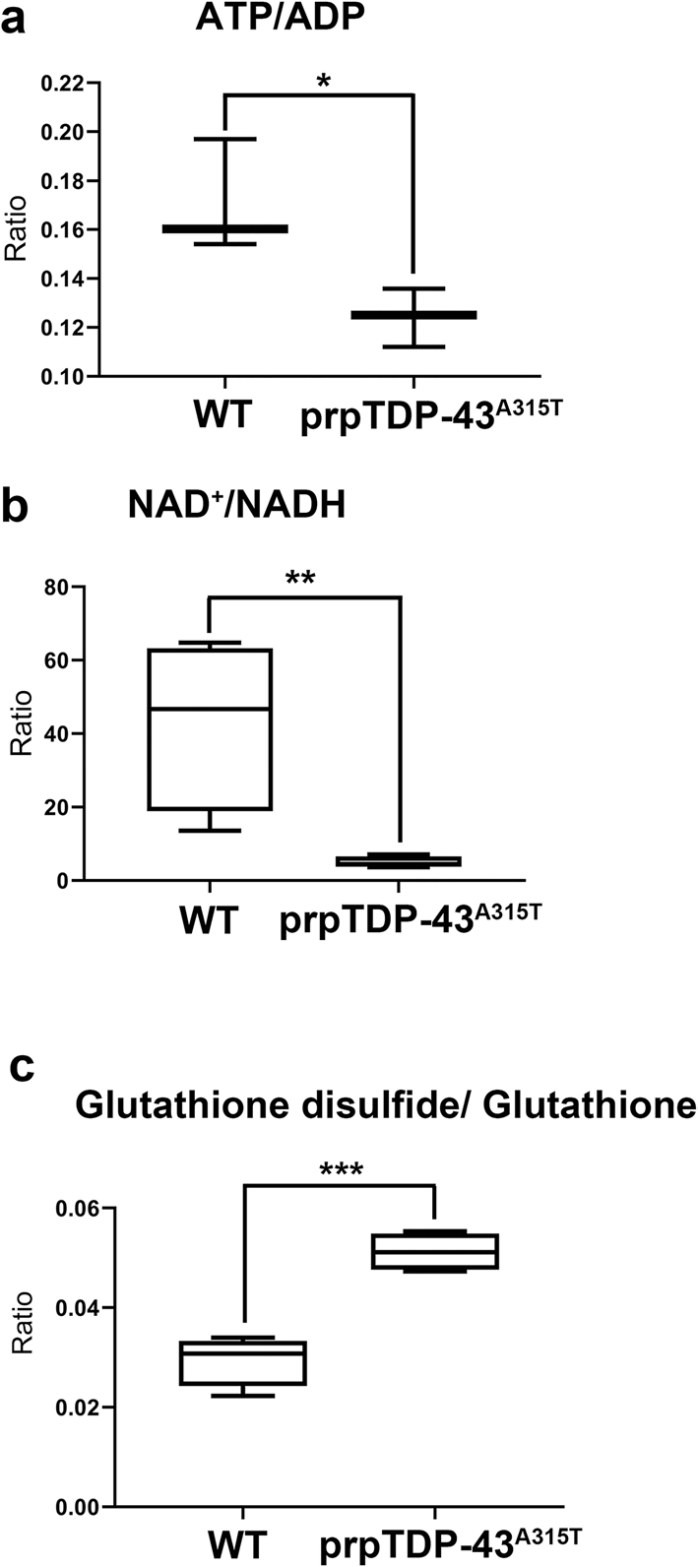
The ratio of key metabolites that are involved in energy homeostasis and oxidative stress are dysregulated in the motor cortex that is diseased due to TDP-43 pathology. (**a**) ratio of ATP/ADP; (**b**) ratio of NAD^+^/NADH and (**c**) ratio of GSSG/GSH.

Detailed analyses revealed striking imbalance especially in the ratio of metabolites that play a key role in energy homeostasis and oxidative stress, such as ATP, NAD^+^ and phosphoenol pyruvate. The ATP/ADP ratio was significantly lower in prpTDP-43^A315T^ motor cortex (WT: 0.17 ± 0.01, *n* = 4; prpTDP-43^A315T^: 0.12 ± 0.006, *n* = 4, p = 0.044, Fig. 3a), and so was NAD^+^/NADH ratio (WT: 42.94 ± 11.73, n = 4; prpTDP-43^A315T^: 4.94 ± 0.77, *n* = 4, p = 0.017, Fig. 3b).

The ratio of GSH and its oxidative derivative GSSG directly corelates with cellular oxidative stress^29^. GSH scavange reactive oxygen species (ROS) and converts into GSSH^30^. Thus, higher GSSG/GSH ratio indicates increased oxidative stress. We find that GSSG/GSH ratio is significantly higher in the motor cortex of prpTDP-43^A315T^ mice even at P90 (WT: 0.02 ± 0.002, *n* = 4; prpTDP-43^A315T^: 0.051 ± 0.001, *n* = 4, p = 0.0005, Fig. 3c). Thus, these altered metabolomic changes and perturbed balance in the motor cortex of prpTDP-43^A315T^ mice suggest a deficit in energy homeostasis and increased oxidative stress, events that are associated with problems with mitochondrial integrity and function.

### UMN culture is an important tool to investigate UMN responses to treatment in vitro

UCHL-eGFP is a reporter mouse line for corticospinal motor neurons (CSMN, a.k.a the UMNs in mice), in which CSMN express eGFP, and CSMN identity of eGFP+ neurons in the layer 5 of the motor cortex is confirmed by retrograde labeling, molecular marker expression and electrophysiological properties^31^. Interestingly, CSMN also retain their eGFP expression in culture. Therefore, UCHL1-eGFP mice allow precise visualization and cellular assessment of UMNs in vitro^31^.

**Figure 4:**
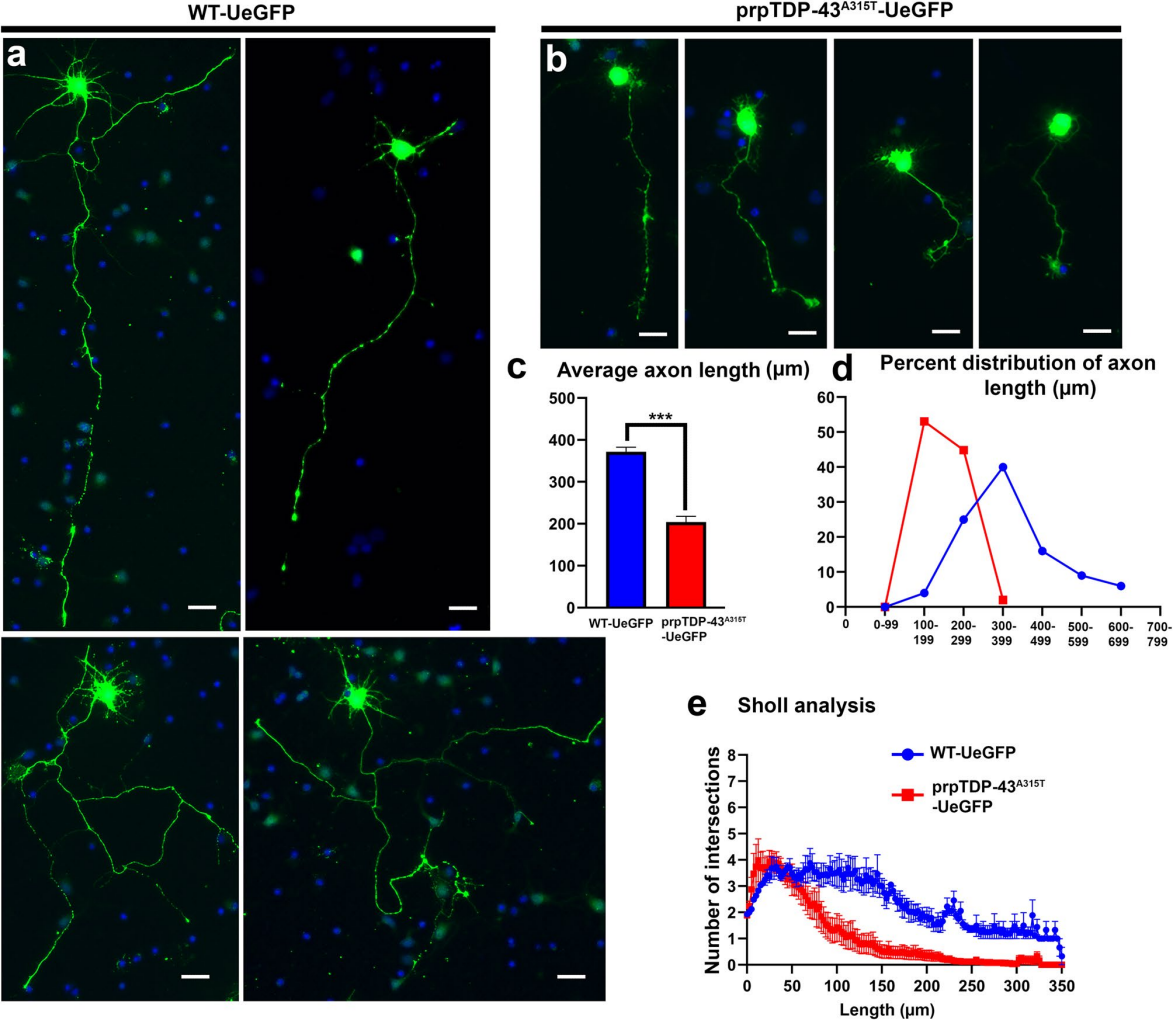
CSMN that express mutated from of human TDP-43^A315T^ extend shorter neurites in vitro. (**a**) representative images of CSMN from WT-UeGFP mouse; (**b**) representative images of CSMN from prpTDP-43^A315T^-UeGFP mouse model of ALS; (**c**) bar graph representation of average neurite length; (**d**) percent distribution of axon length; and (**e**) Sholl analysis of arborization of neurites. Scale bar = 20 µm.

Crossbreeding of UCHL-eGFP with prpTDP-43^A315T^ mouse model of ALS, generated prpTDP-43^A315T^-UeGFP mice (diseased) and WT-UeGFP mice (healthy control). When dissociated cortical cultures are established from these mice, CSMN are distinguished among many different cortical cells and neurons, as they retain their eGFP expression (Fig. 4a,b). Motor cortex is isolated from P3 pups of WT-eGFP and and prpTDP-43^A315T^-UeGFP mice, and dissociated cells are plated on glass coverslips to establish motor cortex cultures in vitro^32^. CSMN of WT-UeGFP mice express eGFP and were healthy with large pyramidal cell bodies, prominent apical dendrites and long axons (Fig. 4a). In contrast, CSMN of prpTDP-43^A315T^-UeGFP mice failed to extend long axons and lacked branching and arborization, even though they retained eGFP expression (Fig. 4b). There was significant reduction in diseased CSMN axon length (WT: 371.7 ± 10.96 μm, n = 3 mice; prpTDP-43^A315T^: 203.9 ± 13.9 μm, n = 3 mice, p = 0.0007, Fig. 4c). Percent distribution of axon length also confirmed that majority of CSMN in prpTDP-43^A315T^-UeGFP mice were shorter when compared to the CSMN of WT-UeGFP mice (Fig. 4d). Diseased CSMN had significantly reduced branching and arborization, as revealed by Sholl analyses (Fig. 4e). Since average CSMN axon length and the extent of neuronal branching and arborization enabled quantitative assessment of differences between healthy and diseased CSMN, these outcome measures were utilized to assess CSMN response to treatment with a cellular precision that was not possible before (Fig. 4c–e).

### Nicotinamide mononucleotide (NMN) improved CSMN health

CSMN display early vulnerability and progressive degeneration in ALS motor cortex and are particularly vulnerable to oxidative stress and energy deficiency^16,17^. Metabolomics analyses revealed NAD^+^ deficiency in the motor cortex. After having established a robust in vitro model for evaluating CSMN health, we next investigated whether increasing NAD^+^ levels could improve their health.

**Figure 5:**
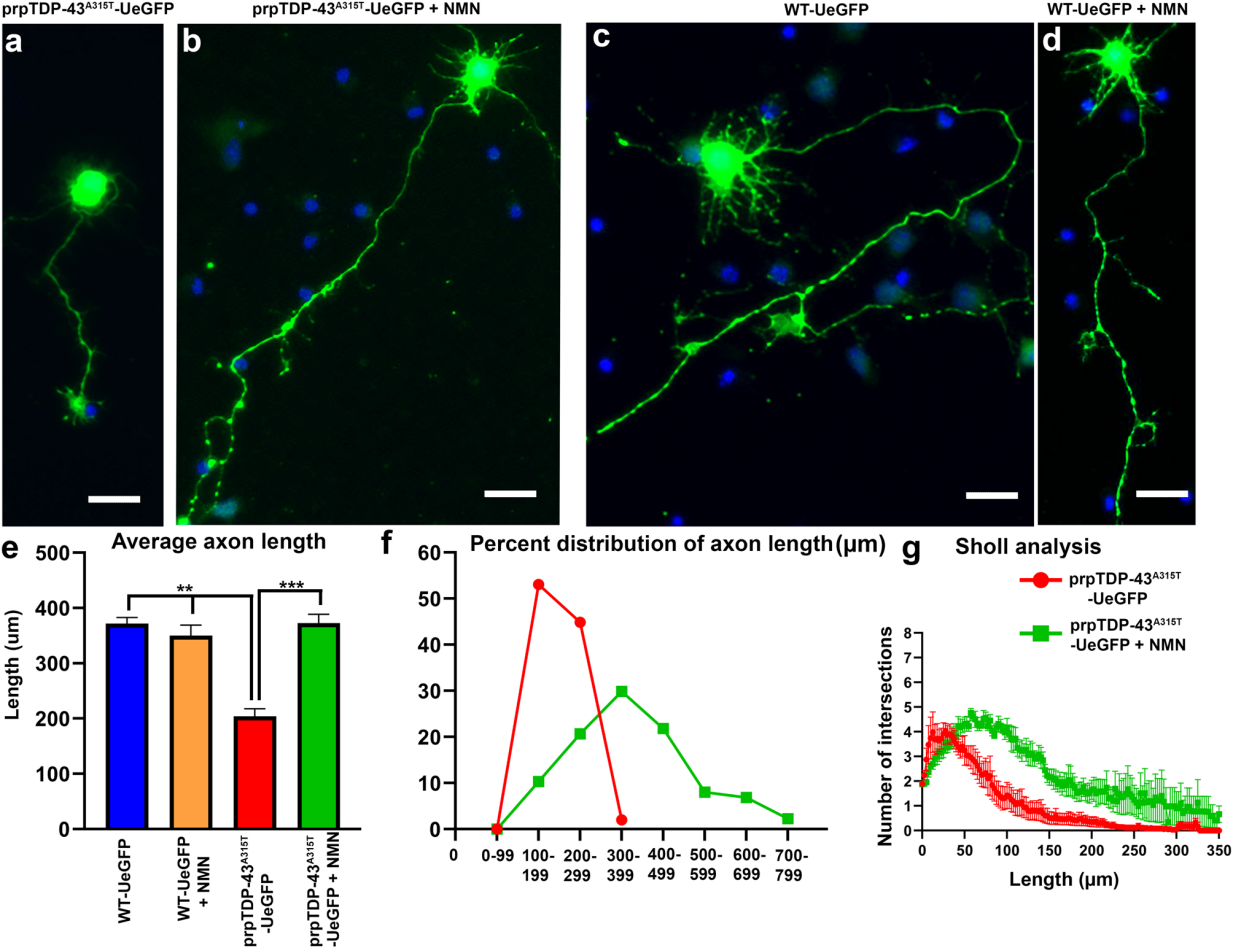
Nicotinamide mononucleotide (NMN) improves the health of CSMN that become diseased due to TDP-43 pathology. (**a**) a representative image of CSMN from prpTDP-43^A315T^-UeGFP mouse; (**b**) a representative image of CSMN from prpTDP-43^A315T^-UeGFP mouse treated with 1 µM NMN; (**c**) a representative image of CSMN from WT-UeGFP mouse; (**d**) a representative image of CSMN from WT-UeGFP mouse treated with NMN. (**e**) Bar graph representation of average neurite length; (**f**) Percent distribution of axon length; and (**g**) Sholl analysis of arborization of neurites. Scale bar = 20 µm.

Since NMN was previously reported to be an NAD^+^ precursor^27^ and NMN led to the production of NAD^+^^26^, we tested the hypothesis that NMN treatment would help maintain the NAD^+^ balance in diseased CSMN, and thus improve their health. NMN was added to the culture medium (1 µM) of both healthy (WT-UeGFP) and diseased (prpTDP-43^A315T^-UeGFP ) motor cultures in vitro, and CSMN responses to treatment was quantitatively assessed by measuring changes in average axon length and the extent of branching and arborization (Fig. 5). CSMN of prpTDP-43^A315T^-UeGFP mice had short axons and did not display branching and arborization (Fig. 5a). However, upon NMN treatment (1 µM), there were significant improvements (Fig. 5b). For example, the average axon length increased in diseased CSMN (prpTDP-43^A315T^-UeGFP: 203.9 ± 13.9 μm, n = 3 mice, prpTDP-43^A315T^-UeGFP + NMN: 372.8 ± 15.8 μm, n = 3 mice; adjusted p value = 0.0002). In addition, the percent distribution of CSMN based on their axon length also revealed an overall and significant shift towards longer axons, such that about 80% of treated CSMN had axons longer than 300 μm, whereas about 90% of untreated CSMN had maximum axon lenth of 200 μm (Fig. 5f).

NIMN treatment was not toxic to WT-UeGFP CSMN (Fig. 5c,d). Regardless of their NMN treatment, they had similar axon lenth and arborization, and were comparable (WT-eGFP: 371.7 ± 10.96 μm, n = 3 mice; WT-eGFP + NMN: 350.3 ± 18.7 μm, n = 3 mice, adjusted p value = 0.753, Fig. 5e).

In addition to axon length, diseased CSMN treated with NMN also displayed improved branching and arborization especially within the 50–200 μm radius, as revealed by Sholl analyses (Fig. 5g, Supplementary Table 1).

### NMN treatment improved mitochondrial stability

**Figure 6:**
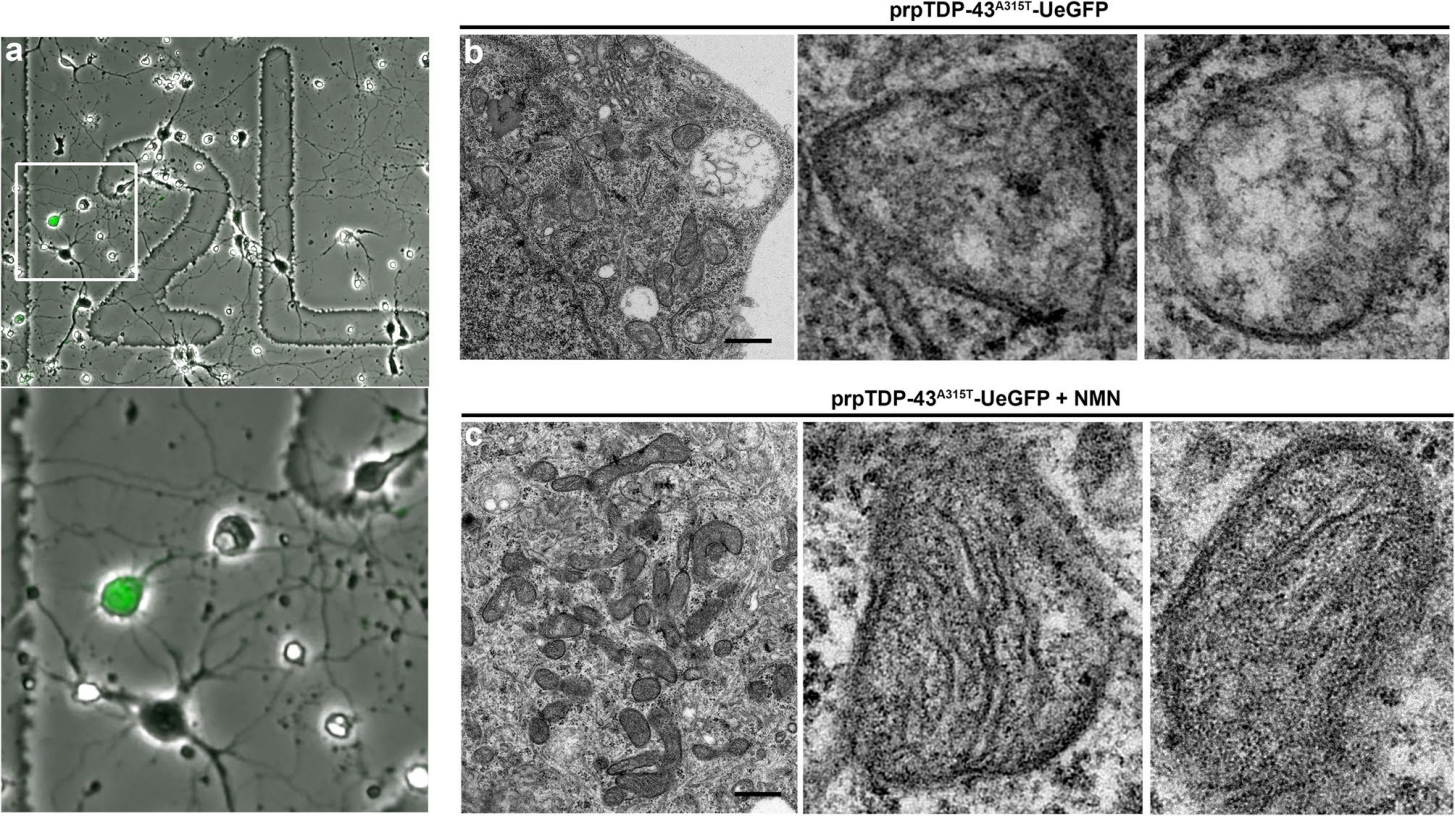
Correlative light electron microscopy shows improvement of mitochondrial ultrastructure upon NMN treatment. (**a**) a representative image of motor cortex neurons cultured on a gridded glass bottom 35 mm dish; boxed area enlarged to highlight presence of GFP expressing CSMN; (**b**) representative electron micrograph of an individual CSMN from prpTDP-43^A315T^-UeGFP mouse showing mitochondrial ultrastructural defects, images of mitochondrion are enlarged to right; and (**c**) representative electron micrograph of an individual CSMN from prpTDP-43^A315T^-UeGFP treated with 1 µM NMN showing improved mitochondrial ultrastructure, images of mitochondrion are enlarged to right. Scale bar = 1 µm.

We used dissociated motor cortex cultures isolated from prpTDP-43^A315T^-UeGFP mice to investigate the basis of NMN-mediated improvement in CSMN health. To visualize the precise ultrastructural changes that occur at an organnelle level, we utilized correlative light electron microscopy (CLEM), on a total of 4 CSMN (untreated (*n* = 2) and NMN treated (*n* = 2)). CSMN were evident with their eGFP expression in vitro (Fig. 6a) and they were prepared for single cell EM analyses to reveal potential changes, especially at the site of mitochondrion. Healthy CSMN from WT-UeGFP mice were used as internal controls with similar treatment paradigms. Most of mitochondria in CSMN of prpTDP-43^A315T^-UeGFP mice had defects especially in their inner mitochondrial membrane; cristae structures were disintegrated and were not detectable (Fig. 6b; CSMN#1: 59%, *n* = 46 mitochondria; CSMN #2: 60%, *n* = 40 mitochondria). However, upon NMN treatment, there were significant improvements in the overall architecture of mitochondria and they had a more intact inner membrane with visible cristae structures. CSMN of prpTDP-43^A315T^-UeGFP mice had fewer diseased mitochondria (Fig. 6c; CSMN #3: 27%, *n* = 55 mitochondria; CSMN # 4: 45%, n = 49 mitochondria). The CLEM results were obtained from 4 independent CSMN isolated from four different mice, revealed the impact of NMN treatment on mitochondrial structure and integrity (Fig. 6b,c).

## Discussion

Mitochondrial problems are mainly studied within the context of energy depletion. However, since mitochondria are also a major site for metabolomic regulation, problems or dysregulation of metabolites are now gaining attention, especially for motor neuron diseases, which display mitochondrial problems as one of the early cellular pathologies. We previously showed the presence of mitochondrial dysfunction as early as P15 in the motor cortex, but more precisely in the CSMN of TDP-43^A315T^ mice^17^, one of the best characterized mouse models of TDP-43 pathology^16,19,33,34^. Numerous other studies, utilizing different animal models^35–37^ or cell lines^38^, all agreed on the presence of mitochondrial problems especially with respect to TDP-43 pathology.

Therefore, we investigated potential metabolomic dysregulation that may occur in the motor cortex of ALS with TDP-43 pathology. We have chosen prpTDP-43^A315T^ mice for our analyses, because this mouse model of TDP-43 pathology was generated based on the *A315T* mutation detected in the *TARDP* gene of ALS patients, who had TDP-43 pathology in their cortex^19^, and because the mouse model strictly recapitulated many aspects of the disease, including progressive CSMN degeneration^16^. Numerous other studies have also chosen this mouse model as one of the best characterized and most informative mouse models for TDP-43 pathology^39–43^. More importantly, when detailed cellular analyses were performed with electron microscopy (EM), exact same cellular problems were detected both in the UMNs of ALS patients with TDP-43 pathology and the CSMN of this mouse model of TDP-43 pathology^16^.

TDP-43 pathology was originally defined with the accumulation of proteins that include phosphorylated TDP-43 (pTDP-43) in the cytoplasm of neurons that undergo degeneration^10^, and this pathology was broadly observed in a wide spectrum of ALS cases^44^. However, recent evidence also reveals TDP-43 accumulations in the nucleus, not only in the cytoplasm^11,12^. Especially in the case of some mutated forms of TDP-43, the protein aggregations are vastly ubiquitinated, but the presence of pTDP-43 is not dominant^14,15,45^. Regardless, patients with TDP-43 mutations have TDP-43 pathology, and the mouse model that have *A315T* mutation has TDP-43 accumulations mostly in the nucleus, have extensive ubiquitinated protein accumulations in the cytoplasm, and based on the antibody used, pTDP-43 are also detected in the cytoplasm^19,46^. Therefore, in line with the current definition of TDP-43 pathology, this mouse model has been considered one of the most reliable models for TDP-43 pathology, especially in the cortex^16,19,39–43^.

Metabolomic dysregulations in the plasma samples isolated from ALS patients were previously reported^47–54^. These studies showed significant alterations in ceramide levels as well as benzoate, creatine and fatty acid metabolisms and sphingomyelins pathways, with respect to ALS progression^49^, and these changes in metabolite levels correlated with patient’s ALS Functional Rating Scale (ALSFRS-R) scores^55^. Likewise, alteration in glucose and lipid metabolisms were reported to contribute to neuronal vulnerability in diseased motor neurons^50^. Components of lipid metabolism such as β-hydroxybutyric acid and medium-chain fatty acids were also found to be dysregulated^56^.

Metabolomic changes begin to reveal the some of the underlying causes of vulnerability in the motor cortex. In this study, we report that a select set of metabolites, such as phosphoenol pyruvate (PEP), glutathione (GSH), 2-hydroxyglutarate (2-HG), and NAD^+^, display significant differences between the healthy and diseased motor cortex with TDP-43 pathology at a symptomatic stage.

Oxidative stress is a prominent physiological feature of ALS, and mechanisms responsible for reducing oxidative stress are compromised^22^. In the motor cortex of ALS patients, glutathione (GSH) levels were reported to be reduced when compared to healthy controls^57^. Likewise, in hSOD1^G93A^ mouse models of ALS, depletion of GSH resulted in degeneration of spinal motor neurons, dysfunctional mitochondria, and neuron loss^58^. GSH is critically important for scavenging reactive oxygen species (ROS) generated by mitochondria^30^, and is also a substrate for antioxidant enzymes that detoxify hydrogen peroxide and lipid peroxides^59^. Importantly, early studies demonstrate a correlation between GSH deficiency and mitochondrial damage^60^. GSH normally exists in its reduced from and is converted into its oxidized form, glutathione disulfide (GSSG), due to oxidative stress. To diminish the effects of oxidative stress, the enzyme glutathione reductase reverts GSSG into GSH. Thus, the ratio of GSH/GSSG is a marker of neuronal oxidative stress and is often used as a marker of cellular toxicity^30^. In resting state, GSSG/GSH ratio were maintained at lower levels, however, in a cell that is experiencing oxidative stress this ratio shifts toward more GSSG^29^. In our study, the motor cortex with TDP-43 pathology displayed increased GSSG/GSH ratio indicating ongoing oxidative stress.

Perturbation of NAD^+^/NADH ratio indicates energy deficiency and is associated with many diseases, including ALS^22^. NAD^+^ accepts H+ released from various substrates and converts into NADH, releasing ATP. A balanced NAD^+^/NADH ratio is critical for continuous generation of ATP. Metabolic dysregulation leads into depletion of NAD^+^ and that in turn affects energy homeostasis. NAD^+^ levels can be restored by compounds such as NR (nicotinamide riboside), NAM (nicotinamide), NA (niacin), and NMN (nicotinamide mononucleotide)^61^. These precursors convert into NAD^+^ that is ready to be used in energy generation pathways. Restoration of NAD^+^ levels had been beneficial and neuroprotective in Alzheimer’s disease and intracerebral hemorrhage^62–65^. Moreover, NAD^+^ enhancement has shown attenuation of astrocyte mediated neurotoxicity in vitro^66,67^. Treatment of hSOD1^G93A^ mouse model of ALS with NR slowed down degeneration of spinal motor neurons and reduced neuroinflammation^68^. In an in vitro model of TDP-43 deficiency in spinal motor neurons, loss of TDP-43 is accompanied by axonal defects. Lack of TDP-43 leads to perturbed transcriptome in axon and dendrites and dysregulated transcripts code for genes that are key component of mRNA translation, cytoskeleton, and mitochondria mediated energy production and metabolism^35^.

Building evidence, including the current study, suggests that mitochondria mediated metabolite disturbances are emerging as prevalent feature in ALS, and restoration of NAD^+^ levels by NAD+ precursor compounds could in fact be a potential therapeutic strategy for improving neuron health. Therefore, we tested whether NMN treatment would help improve the health of CSMN that are diseased due to TDP-43 pathology.

In an effort to investigate whether NMN treatment would have an impact on the health of UMNs diseased with TDP-43 pathology, we utilized dissociated motor cortex cultures established from prpTDP-43^A315T^-UeGFP mice, in which CSMN are genetically labeled with eGFP expression that is stable and long-lasting, and when in culture retain their CSMN identity and eGFP expression. These diseased reporter mice were exceptionally valuable for directly assessing the cellular responses of healthy and diseased CSMN to treatments in vitro and in vivo^69–71^*.* Here, we utilized them to investigate their response to NMN treatment. We find that acute and short-term treatment of diseased CSMN with NMN not only improve their neuronal health, but also help mitochondria to regain the integrity of their inner membrane. Our results reveal early metabolomic dysfunction in the motor cortex of prpTDP-43^A315T^ mice and begin to suggest that reestablishing the perturbed balance may help UMNs regain their health and stability.

Even though long-term exposure to NMN may not be favorable for axonal stability or neuron health due to its interaction with Sterile alpha and Toll/interleukin-1 receptor motif-containing 1 (SARM1), and by modulating its activity^72,73^, maintaining NAD^+^/ NADH balance appears to be an important contributor to UMN health, especially the ones that are diseased due to TDP-43 pathology. Therefore, our results suggest the unique importance of understanding the metabolomic problems that occur early in the diseased motor cortex, and encourages for the development of novel therapeutic strategies by reestablishing the perturbed NAD^+^ levels in the brain.

## Conclusions

Metabolomic defects begin to occur early in the motor cortex with TDP-43 pathology. There is altered energy balance and reduced levels of ATP. Since metabolomic analyses reveal reduced levels of NAD^+^ as a potential converging outcome of altered metabolomic imbalance, NMN treatment is applied to upregulate NAD^+^ levels and to investigate the relationship between increased NAD^+^ and CSMN health. Our findings, which utilize a novel CSMN reporter line with TDP-43 pathology, not only reveal the presence of metabolomic defects at an early stage of the disease, but also suggest that proper modulation of metabolomic imbalance could offer therapeutic strategies to improve the integrity of mitochondria, and thus the health of diseased UMNs.

## Materials and methods

### Animals

All animal experiments were performed in compliance with the standards set by National Institutes of Health (NIH) and were reviewed and approved by the Northwestern University Institutional Animal Care and Use committee (IACUC approval number # IS00004844). All experiments were conducted in compliance with the ARRIVE guidelines. The following mouse strains were used in this study: prp-TDP-43^A315T^ (procured from Jackson Laboratory, stock no. 010700), and UCHL1-eGFP (generated by the Ozdinler Laboratory at Northwestern Targeted Mutagenesis Core Facility, now also available at Jackson Laboratory, stock no. 022476). Gastrointestinal obstruction have been reported in the prp-TDP-43^A315T^ mouse line^74^. However, feeding these mice with high fat jelly diet prevented their gastrointestinal problems and their death was reported to be due to TDP-43 pathology mediated degeneration, and not due to gastrointestinal problems^75,76^. In this study, all TDP-43 mouse models were fed with the jelly diet. Hemizygous UCHL1-eGFP females were bred to hemizygous prp-TDP-43^A315T^ males to generate prp-TDP-43^A315T^-UeGFP mice. All the mice used in this study were on C57/BL6 background and were P90 at the time of analyses. Animals were housed at a controlled temperature (23 °C) and controlled light–dark cycle (12–12 h), with free access to water and food^16,17^.

### Tissue preparation and metabolite extraction

Mice were deeply anesthetized with intraperitoneal injection of Ketamine (90 mg/kg, and Xylazine (10 mg/kg; Fort Dodge Animal Health, Fort Dodge, IA, USA)^16^. Intact brain was removed and motor cortex was dissected out, weighed and immediately frozen in liquid nitrogen. The tissue was homogenized in 1 ml chilled 80% chromatography grade methanol and vigorously vortexed three times. 200 µL tissue homogenate was mixed into a tube pre-added with 800 µL of ice-cold methanol/water 80% (vol/vol) followed by vortexing rigorously for 1 min, and then centrifuge at ~ 20,160*g* for 15 min in a refrigerated centrifuge. The metabolite-containing supernatant was separated into a fresh tube and stored at − 80 °C until metabolite profiling was performed^77^.

### Method for sample reconstitution after extraction

Extraction solution was dried using SpeedVac. 50% acetonitrile was added to the tube for reconstitution following by vigorously shaking for 30 s. Samples solution was then centrifuged for 15 min @ 20,000*g*, 4 °C. Supernatant was collected for LCMS analysis^77^.

### Hydrophilic metabolites profiling

Comprehensive metabolomics was performed as described earlier^77^. Briefly, samples were analyzed by High-Performance Liquid Chromatography and High-Resolution Mass Spectrometry and Tandem Mass Spectrometry (HPLC–MS/MS). Specifically, system consisted of a Thermo Q-Exactive in line with an electrospray source and an Ultimate3000 (Thermo) series HPLC consisting of a binary pump, degasser, and auto-sampler outfitted with a Xbridge Amide column (Waters; dimensions of 4.6 mm × 100 mm and a 3.5 µm particle size). The mobile phase A contained 95% (vol/vol) water, 5% (vol/vol) acetonitrile, 20 mM ammonium hydroxide, 20 mM ammonium acetate, pH = 9.0; B was 100% Acetonitrile. The gradient was as following: 0 min, 15% A; 2.5 min, 30% A; 7 min, 43% A; 16 min, 62% A; 16.1–18 min, 75% A; 18–25 min, 15% A with a flow rate of 400 μL/min. The capillary of the ESI source was set to 275 °C, with sheath gas at 45 arbitrary units, auxiliary gas at 5 arbitrary units and the spray voltage at 4.0 kV. In positive/negative polarity switching mode, an *m*/*z* scan range from 70 to 850 was chosen and MS1 data was collected at a resolution of 70,000. The automatic gain control (AGC) target was set at 1 × 10^6^ and the maximum injection time was 200 ms. The top 5 precursor ions were subsequently fragmented, in a data-dependent manner, using the higher energy collisional dissociation (HCD) cell set to 30% normalized collision energy in MS2 at a resolution power of 17,500. Data acquisition and analysis were carried out by Xcalibur 4.1 software and Tracefinder 4.1 software, respectively (both from Thermo Fisher Scientific).

### Correlative light electron microscopy (CLEM)

Mixed cortical cultures from prpTDP-43^A315T^ mice were plated on a gridded glass coverslip bottom petridishes (MetTek) and cultured for 3 DIV either in SFM alone or treated with 1 µM NMN (Sigma). Correlative Light Electron microscopy (CLEM) was performed as describes previously^78^. After fixing with 2% PFA and 0.5% glutaraldehyde for 10 min at room temperature, GFP+ CSMN were identified under epifluorescent microscope, and their location on the grid marked before processing for EM analysis. Cells were further post-fixed in 2% PFA and 2% glutaraldehyde for 10 min at room temperature and 50 min at 4 °C. Cells were washed with 0.12 M phosphate buffer pH 7.4 and treated with 1% osmium tetroxide (EMS) and 1.5% potassium ferrocyanide (Sigma) dissolved in 0.12 M phosphate buffer. Cells were dehydrated by subjecting them to ascending series of alcohol 50%, 70%, 80%, 90% and 100% followed by treatment with Epoxy resin for 24 h. The grids were mounted on resin blocks and cured at 65 °C for 3 days. The blocks were trimmed to contain only CSMN, before proceeding to ultra-thin sectioning. Resin blocks were ultra thin sectioned on a Leica Ultracut UC6 ultramicrotome (Leica Inc., Nussloch, Germany). 70 nm thin sections were collected on 200-mesh copper–palladium grids. Ultra-thin sections were counterstained on a drop of UranyLess solution (Electron Microscopy Sciences, Hatfield, PA, USA) and 0.2% lead citrate. Grids were examined on FEI Tecnai Spirit G2 TEM (FEI company, Hillsboro, OR, USA), and digital images were captured on a FEI Eagle camera.

### Motor cortex cultures

P3 motor cortices isolated from WT-UeGFP, and prpTDP-43^A315T^–UeGFP mice, dissected, dissociated, and cultured on glass coverslips (4 × 10^4^ cells per 18 mm diameter coverslip, Fisherbrand) coated with poly-L-lysine (10 mg/mL, Sigma). Neurons were cultured in serum-free medium [SFM; 0.034 mg/L BSA, 1 mM L-glutamine, 25 U/mL penicillin, 0.025 mg/mL streptomycin, 35 mM glucose, and 0.5% B27 in Neurobasal-A medium (Life Technologies)] in a humidified tissue culture incubator in the presence of 5% CO_2_ at 37 °C as previously described^28^. NMN (1 µM) was added at the start of the culture. Cultures were fixed after 3 days in vitro.

### Immunocytochemistry

Anti-GFP (1:1000, Abcam) antibody was applied in blocking solution (PBS, 0.05% BSA, 2% FBS, 1% Triton X-100, and 0.1% saponin) for 2 h at room temperature. After incubation, and washes with PBS, appropriate secondary fluorescent antibodies (1:500, AlexaFluor-conjugated, Invitrogen) were added to the blocking solution at room temperature for 2 h in the dark. Nuclei were counterstained with DAPI.

### Data analysis

#### Measurement of axon length

CSMN from WT-UeGFP, and prp-TDP-43^A315T^-UeGFP mice (*n* = 3) were culture in vitro for 3 days. Immunocytochemistry was performed with eGFP to enhance visualization of GFP+ neurons. Images were acquired using epifluorescent microscope. Neurite length was measure using FIJI ImageJ software. CSMN processes were traced using the Simple Neurite Tracer plugin from FIJI (NIH), which enables semi-autonomous tracing of neuron morphology. The longest neurite was selected to measure the length of the axon in µm.

#### Sholl analyses

The aggregation of the neurite tracings centered at the soma generates a profile available for Sholl analysis to quantify number of intersections at 5 µm radius intervals for each neuron included in analysis. Minimum 15 neurons per treatment from 3 independent experiments were used for analysis.

### Statistics

Statistical analyses were performed using Prism software (GraphPad Software Inc., La Jolla, CA, USA)^16^. For each quantification at least *n* = 3 mice were used for each genotype and group. D’Agostino and Pearson normality tests were performed on all data sets. Student’s *t* test was used to determine statistical differences between two experimental groups depending on the genotype, experimental conditions and the disease group. One way ANOVA was used for analysis of more than two groups. Data are shown as mean ± SEM of at least three replicates and are representative of three independent experiments unless otherwise stated and statistically significant differences were taken at *p* < 0.05, and *p* values are reported in the text.

## Supplementary Information

Supplementary Information 1.

Supplementary Information 2.
