# Supplementary material for: Mitochondrial dysregulation occurs early in ALS motor cortex with TDP-43 pathology and suggests maintaining NAD+ balance as a therapeutic strategy
Source: Sci Rep. 2022 Mar 11;12:4287. doi: 10.1038/s41598-022-08068-5 (PMC8917163; doi:10.1038/s41598-022-08068-5)
Supplement: Supplementary file 1 — Supplementary Information 1. [file 41598_2022_8068_MOESM1_ESM.pdf]

		Ozd-Muk-20190207-01.raw	Ozd-Muk-20190207-02.raw	Ozd-Muk-20190207-03.raw	Ozd-Muk-20190207-04.raw	Ozd-Muk-20190207-05.raw	Ozd-Muk-20190207-06.raw	Ozd-Muk-20190207-07.raw	Ozd-Muk-20190207-08.raw
**Total Iron Count (another option for normalization)**	**TIC**	6.0319E+10	6.2993E+10	6.3596E+10	6.1500E+10	5.7309E+10	6.1846E+10	6.5871E+10	7.0917E+10
	**KEGG ID**	**WT 1**	**WT 2**	**WT 3**	**WT 4**	**TDP-43-1**	**TDP-43-2**	**TDP-43-3**	**TDP-43-4**
**2-HG**	**C02630**	233,338,781	265,339,744	251,461,784	252,495,520	230,836,168	265,801,089	297,563,156	257,662,936
**3-phospho-serine**	**C01005**	15,029	88,400	73,320	50,071	79,189	108,074	104,553	298,167
**5-methylcytosine**	**C02376**	198,089	168,755	240,618	261,241	102,704	72,453	175,162	293,213
**6-O-methylguanine**	**n/a**	0	0	0	0	0	0	0	0
**6-phosphogluconic acid**	**C00345**	512,411	857,435	708,932	626,506	378,192	329,319	908,180	2,288,451
**7-methylguanine**	**C02242**	423,864	545,983	490,371	441,151	341,059	504,734	583,955	1,017,938
**acetoacetic acid**	**C00164**	0	1,735,956	1,870,857	1,953,313	1,527,718	1,006,422	0	0
**acetylcholine+**	**C01996**	289,275,123	232,697,712	275,841,093	295,380,184	323,835,041	328,311,539	354,141,497	369,806,344
**aconitic acid**	**C00417-C02341**	1,736,130	2,241,484	2,300,695	0	16,090,504	26,938,254	29,959,008	33,356,585
**adenine**	**C00147**	104,691,720	152,149,296	159,164,754	154,795,702	67,522,308	134,106,223	110,346,876	203,827,297
**adenosine**	**C00212**	992,415,441	1,124,785,734	1,125,453,604	1,260,125,577	798,932,402	1,020,875,733	1,311,831,642	1,337,464,830
**adenylosuccinic acid**	**C03794**	39,818	19,946	61,756	32,916	49,755	14,604	32,730	14,920
**AICA ribonucleotide**	**C04677**	0	0	0	0	0	0	0	0
**a-KG**	**C00026**	28,101,464	33,401,504	33,955,774	35,676,423	34,198,407	39,063,463	45,885,122	56,207,732
**allantoin**	**C01551**	3,705,295	6,577,713	5,735,487	4,366,483	5,851,460	6,234,188	10,053,635	14,729,889
**arginine**	**C02385**	762,022,977	761,616,718	796,023,072	799,412,500	471,315,088	831,196,675	927,391,306	992,455,004
**asparagine**	**C16438**	8,010,935	12,265,908	12,623,470	10,257,601	9,436,676	12,455,231	15,676,921	13,731,604
**aspartic acid**	**C16433**	366,377,134	503,623,316	505,995,339	425,249,490	494,060,036	452,376,861	522,235,570	454,142,285
**ATP / dGTP**	**C00002 / C00286**	3,652,196	3,768,264	2,777,869	2,840,176	2,670,874	2,586,805	3,070,322	6,370,153
**betaine**	**C00719**	600,124,290	681,036,103	700,241,088	669,998,493	670,427,247	859,095,592	962,599,569	825,980,326
**carnitine+**	**C00487**	1,967,138,524	2,388,339,546	2,257,498,389	1,976,161,901	1,790,668,487	2,758,203,294	2,482,327,396	2,994,299,444
**CDP**	**C00112**	227,148	121,272	102,819	54,420	122,791	0	66,784	44,392
**choline+**	**C00114**	3,138,556,733	4,171,365,520	4,541,506,659	4,114,004,935	3,768,779,936	4,310,236,285	5,037,893,913	4,709,671,026
**citraconic acid**	**C02226**	0	0	0	0	0	0	0	0
**citrate / citrate(iso)**	**C00158 / C00311**	663,758,375	839,603,834	927,206,912	875,909,182	933,029,506	985,983,705	1,171,294,715	1,361,714,533
**citrulline**	**C00327**	8,081,443	9,540,408	10,412,592	8,061,704	6,905,605	8,572,293	8,964,303	11,122,377
**CMP**	**C00055**	4,073,333	3,175,143	3,412,307	2,771,984	3,724,520	1,850,820	4,396,725	2,223,002
**creatine**	**C00300**	13,447,444,932	15,795,616,284	15,129,010,813	15,055,397,915	13,128,616,848	14,770,073,842	15,854,576,512	17,783,164,417
**Creatinine**	**C00791**	89,435,655	123,412,056	109,558,458	94,083,401	42,823,184	121,289,550	154,215,440	182,075,803
**CTP**	**C00063**	0	0	0	0	0	0	0	9,884
**cystathionine**	**C00542**	1,875,986	1,333,870	1,428,253	1,865,283	940,383	2,362,384	2,503,396	9,387,183
**cysteine**	**C00736-C00097**	2,439,433	1,903,343	3,043,142	4,006,167	2,360,352	2,083,096	3,959,283	2,924,349
**cystine**	**C01420**	571,459	281,102	518,626	587,694	390,378	235,526	752,245	331,238
**cytidine**	**C00475**	9,142,418	15,990,816	16,812,927	14,723,832	7,460,931	13,539,232	14,073,589	27,695,508
**cytosine**	**C00380**	528,976	660,425	751,427	582,589	512,493	691,669	813,473	1,227,657
**dADP**	**C00206**	0	0	0	0	0	0	0	0
**dAMP**	**C00360**	515,287	166,546	374,940	674,087	807,870	843,673	522,384	234,044
**D-arabinono-1,4-lactone**	**C00652**	105,913,981	114,015,451	124,381,975	106,897,802	54,916,818	63,265,218	73,591,195	101,436,795
**dATP**	**C00131**	0	0	0	0	0	0	0	0
**dCMP**	**C00239**	0	0	0	0	0	0	0	0
**dCTP**	**C00458**	0	0	0	0	0	0	0	0
**deoxyguanosine**	**C00330**	992,448,792	1,124,785,734	1,125,453,604	1,260,253,890	799,025,488	1,020,895,363	1,312,271,414	1,336,877,616
**deoxyuridine**	**C00526**	1,143,121	1,413,785	2,744,112	1,566,750	907,902	1,104,869	1,859,937	7,390,189
**D-glucosamine-6-phosphate**	**C00352**	188,286	26,034	57,160	0	39,267	27,548	21,250	15,590
**dihydroorotate**	**C00337**	26,426	0	26,657	25,290	0	0	28,633	0
**DL-DOPA**	**C00355**	46,526	18,990	0	30,143	114,802	97,439	107,549	22,698
**D-sedoheptulose-7-phosphate**	**C05382**	963,746	1,473,730	1,474,940	1,341,985	1,084,859	1,506,380	1,693,140	1,647,419
**dTMP**	**C00364**	0	0	0	0	0	0	0	0
**dTTP**	**C00459**	0	0	0	0	0	0	0	0
**dUMP**	**C00365**	0	0	0	0	0	0	0	0
**epinephrine**	**C00788**	22,470	11,208	37,825	32,796	32,614	17,236	12,135	0
**FAD**	**C00016**	0	78,034	58,322	0	74,175	146,230	108,897	122,305
**FMN**	**C00061**	0	0	0	0	0	0	0	0
**fumaric acid**	**C00122**	85,907,695	96,537,411	98,654,595	129,170,802	103,062,224	97,023,778	161,443,425	124,825,871
**GABA**	**C00334**	1,363,841,197	1,546,970,627	1,556,689,559	1,502,112,809	1,338,257,441	1,667,460,177	1,591,375,639	2,054,661,149
**GDP**	**C00035**	6,852,934	8,255,043	7,477,870	8,870,204	8,649,455	8,505,338	12,038,876	12,950,516
**glucono-lactone**	**C00198**	2,056,628	2,513,168	2,512,305	1,801,774	679,362	1,061,552	1,604,443	1,402,437
**glutamine**	**C00303**	1,633,413,569	1,951,382,025	2,002,231,354	1,855,567,842	1,690,279,279	2,016,100,037	2,466,110,458	5,601,767,768
**glutaminic acid**	**C00302**	1,827,527,809	2,331,782,146	2,293,498,839	2,147,148,210	1,844,285,539	2,160,746,918	2,512,856,981	2,781,540,499
**glutathione**	**C00051**	239,488,167	321,450,379	320,557,628	314,281,824	228,175,887	304,122,948	342,906,189	428,762,181
**glutathione disulfide**	**C00127**	7,225,045	10,924,470	10,045,570	7,001,304	10,792,385	16,827,797	18,351,895	20,873,638
**glyceric acid**	**C00258**	8,202,965	10,973,816	9,346,125	8,446,219	6,626,909	7,235,080	8,784,236	8,791,267
**glycine**	**C00037**	15,491,759	19,640,216	20,568,309	16,238,240	19,051,738	23,373,022	33,752,411	28,449,726
**GMP**	**C00144**	36,006,512	44,033,239	46,833,505	39,092,226	35,225,021	40,650,320	47,376,047	49,522,059
**GTP**	**C00044**	820,048	761,809	878,095	927,090	841,001	859,536	860,393	1,525,558
**guanine**	**C00242**	265,581	489,519	469,447	309,859	624,986	749,374	872,101	981,257
**guanosine**	**C00387**	7,596,991	9,431,155	10,460,775	11,523,086	4,107,836	8,947,820	7,776,504	15,891,148
**histamine**	**C00388**	3,950,619	3,864,226	3,490,019	4,057,121	3,331,630	3,286,096	3,809,032	9,127,603
**histidine**	**C00768**	447,693,671	524,541,240	559,897,674	557,048,235	406,227,182	482,285,591	596,402,001	886,635,570
**homocysteine**	**C05330**	45,977	37,519	0	0	0	103,790	28,388	364,176
**hydroxyphenyllactic acid**	**C03672**	15,175,351	18,977,340	19,125,536	17,798,966	12,220,058	14,011,719	11,764,725	16,100,138
**hypoxanthine**	**C00262**	90,774,843	121,234,392	148,179,816	115,559,035	150,934,623	193,117,196	180,587,476	252,634,853
**IDP**	**C00104**	0	0	0	0	0	0	0	0
**IMP**	**C00130**	28,852,690	19,008,037	21,872,390	16,545,855	19,488,222	8,866,993	15,757,709	14,740,789
**inosine**	**C00294**	71,620,648	78,873,580	105,965,339	85,564,073	57,637,121	91,180,156	80,542,977	158,721,315
**kynurenine**	**C01718**	55,300	66,031	89,240	107,019	53,478	157,041	130,751	2,202,793
**lactic acid**	**C01432**	3,957,481,241	4,411,176,425	4,462,737,208	4,271,741,127	2,988,261,810	4,563,084,108	5,152,564,524	5,884,212,838
**L-arginino-succinate**	**C03406**	1,579,831	2,196,708	2,299,970	2,270,899	1,717,292	1,983,410	2,834,528	3,017,605
**levulinic acid**	**n/a**	0	0	1,566,139	904,323	872,914	3,414,441	0	1,702,789
**L-NMMA**	**C03884**	4,629,646	4,397,463	4,621,045	4,536,677	3,073,488	4,011,370	7,399,016	6,353,360
**lysine**	**C00047/C00739**	101,130,992	102,918,446	107,093,456	89,251,546	85,287,528	108,435,452	154,196,485	144,324,520
**maleic acid**	**C01384**	0	0	0	1,525,338	0	0	0	0
**malic acid**	**C00711**	1,009,279	11,705,340	13,130,203	14,544,791	13,061,583	12,346,241	14,697,945	17,903,667
**methionine**	**C00073**	35,657,506	43,712,720	46,831,093	40,581,132	33,253,560	43,078,961	52,965,012	53,502,831
**methylglyoxal**	**C00546**	262,884	275,072	214,675	345,795	77,655	124,486	186,502	213,975
**mevalonic acid**	**C00418**	502,693	780,677	595,215	883,762	487,127	658,603	604,832	681,098
**N-acetylaspartic acid**	**C01042**	9,376,816,824	10,455,225,059	10,608,157,311	9,745,007,744	8,432,551,632	9,640,666,620	10,967,827,441	12,419,167,663

**N-acetylaspartylglutamic acid**	**C12270**	2,559,805	3,999,847	4,205,742	3,045,890	1,962,251	2,235,445	2,375,569	5,200,647
**N-acetyl-DL-alanine**	**n/a**	4,034,307	5,235,471	5,271,576	5,544,716	3,352,692	4,772,855	4,727,454	6,446,052
**NAD+**	**C00003**	3,168,963	4,810,802	4,354,923	4,012,039	2,804,783	4,640,501	3,256,800	5,378,023
**NADH**	**C00004**	54,042	353,948	67,254	115,307	558,486	652,423	816,487	1,472,849
**NADP+**	**C00006**	539,227	694,326	468,541	621,283	499,468	616,298	723,903	782,197
**NADPH**	**C00005**	0	48,727	62,286	70,996	77,279	37,576	17,052	0
**N-carbamoyl-L-aspartate**	**C00438**	0	0	0	0	0	0	0	0
**nicotinamide**	**C00153**	291,498,477	321,677,540	359,812,861	320,817,228	329,115,310	332,960,403	488,201,581	450,000,632
**N-methyltryptamine**	**C06213**	6,217	6,904	7,365	14,619	11,572	8,780	0	17,573
**norepinephrine**	**C00547**	93,681	115,308	163,488	112,133	118,619	92,925	36,698	175,863
**ornithine**	**C01602**	4,994,856	8,267,196	8,971,962	6,012,984	4,138,248	6,254,713	6,002,810	6,057,181
**orotic acid**	**C00295**	2,861,654	3,502,861	3,487,754	3,792,731	2,373,319	3,291,925	3,205,375	3,024,776
**orotidylic acid**	**C01103**	0	0	0	0	0	0	0	0
**oxalacetic acid**	**C00036**	139,186	126,127	101,954	78,926	149,756	285,787	115,965	0
**pantothenic acid**	**C00864**	53,393,629	66,355,345	65,885,127	55,048,694	73,273,236	85,198,895	83,310,670	214,224,065
**phenethylamine**	**C05332**	379,396	450,463	451,119	395,152	401,178	430,278	470,857	430,360
**phenylalanine**	**C02057**	49,990,483	69,161,322	74,909,280	65,532,125	51,148,271	76,618,846	89,549,653	208,630,017
**phosphocreatine**	**C02305**	579,884	647,778	607,525	472,663	942,220	726,778	480,346	724,020
**phosphoenolpyruvate**	**C00074**	57,087	1,116,434	854,044	297,910	2,606,547	3,477,722	5,992,997	13,782,009
**phosphorylcholine+**	**C00588**	295,268,296	460,821,113	463,299,857	417,720,937	370,723,689	555,882,039	856,753,358	957,869,704
**proline**	**C16435**	119,281,141	161,303,433	159,997,543	132,959,750	94,079,947	132,237,987	158,336,458	264,889,816
**PRPP**	**C00119**	0	0	0	0	0	0	0	0
**putrescine**	**C00134**	96,407	29,473	88,728	20,483	85,327	195,975	18,626	56,778
**pyruvic acid**	**C00022**	28,202,922	22,161,757	16,837,752	25,994,696	7,413,031	12,269,335	16,392,178	25,300,866
**S-adenosyl-L-homocysteine**	**C00021**	236,995	481,490	470,563	360,824	219,725	695,482	419,951	1,318,196
**S-adenosyl-L-methionine+**	**C00019**	0	187,129	229,507	339,872	214,550	178,148	392,238	230,936
**serine**	**C00716**	69,917,271	80,914,522	86,860,369	80,132,323	92,850,197	102,431,269	115,571,550	97,559,642
**serotonin**	**C00780**	73,031	94,425	44,367	43,799	70,350	24,880	24,623	39,563
**spermidine**	**C00315**	102,508,961	93,394,055	115,278,143	159,406,986	103,389,583	98,559,813	91,751,751	85,011,415
**spermine**	**C00750**	19,339,643	15,085,037	21,045,199	14,978,731	22,931,370	19,536,470	27,647,714	19,933,526
**succinic acid**	**C00042**	1,289,215,393	1,937,908,967	1,837,071,583	1,386,643,010	1,294,558,225	2,072,764,686	1,765,155,125	2,959,824,088
**thiamine+**	**C00378**	12,298,582	14,235,088	15,393,670	12,610,923	12,060,207	16,239,474	18,914,866	17,606,317
**thymidine**	**C00214**	944,639	916,254	1,910,454	1,172,623	694,026	987,815	1,377,739	5,515,359
**thymine**	**C00178**	704,449	800,739	1,383,199	767,767	637,758	725,637	1,011,800	3,329,126
**tryptamine**	**C00398**	10,260	21,905	11,938	12,986	35,252	27,884	21,484	34,875
**tryptophan**	**C00806**	10,341,372	15,910,967	16,057,172	14,842,478	8,947,045	15,844,346	22,069,618	46,459,970
**tyramine**	**C00483**	575,992	551,200	574,717	304,227	999,017	405,305	574,040	316,755
**tyrosine**	**C01536**	28,878,040	38,855,524	38,771,718	34,511,169	28,369,355	37,622,702	31,227,728	46,060,699
**UDP**	**C00015**	419,847	553,580	528,919	303,516	582,661	464,010	662,434	1,052,398
**UDP-D-glucose**	**C00029**	1,270,312	1,990,118	1,763,501	1,530,374	1,022,994	1,711,995	1,936,192	1,814,802
**UMP**	**C00105**	3,486,271	3,135,127	3,269,653	2,635,173	3,651,848	2,392,191	4,393,651	2,547,084
**uracil**	**C00106**	24,178,266	38,620,665	42,453,033	35,097,557	23,371,051	42,668,665	35,265,332	73,441,253
**uric acid**	**C00366**	1,506,946	2,252,259	2,464,075	1,676,617	2,241,740	3,300,160	3,460,686	6,580,823
**uridine**	**C00299**	22,964,308	34,608,322	37,530,621	32,052,382	20,401,569	38,632,363	38,619,704	63,382,303
**UTP**	**C00075**	15,980	18,114	0	0	0	0	0	0
**valine**	**C16436**	13,412,245	16,488,450	12,761,287	9,898,551	7,286,406	14,321,239	20,763,858	24,118,125
**xanthine**	**C00385**	41,260,080	50,821,648	49,220,920	55,564,204	61,167,998	68,555,454	75,244,405	65,543,503
**XMP**	**C00655**	0	0	34,361	18,427	0	6,692	0	74,811
