# Supplementary material for: Mitochondrial dysregulation occurs early in ALS motor cortex with TDP-43 pathology and suggests maintaining NAD+ balance as a therapeutic strategy
Source: Sci Rep. 2022 Mar 11;12:4287. doi: 10.1038/s41598-022-08068-5 (PMC8917163; doi:10.1038/s41598-022-08068-5)
Supplement: Supplementary file 2 — Supplementary Information 2. [file 41598_2022_8068_MOESM2_ESM.docx]

Supplemental Table: The list of metabolites detected in the motor cortex of WT and prpTDP-43^A315T^ mice
